# Antiviral Phosphorodiamidate Morpholino Oligomers are Protective against Chikungunya Virus Infection on Cell-based and Murine Models

**DOI:** 10.1038/srep12727

**Published:** 2015-07-30

**Authors:** Shirley Lam, Huixin Chen, Caiyun Karen Chen, Nyo Min, Justin Jang Hann Chu

**Affiliations:** 1 Laboratory of Molecular RNA Virology and Antiviral Strategies, Department of Microbiology, Yong Loo Lin School of Medicine, National University Health System, National University of Singapore, Singapore.

## Abstract

Chikungunya virus (CHIKV) infection in human is associated with debilitating and persistent arthralgia and arthritis. Currently, there is no specific vaccine or effective antiviral available. Anti-CHIKV Phosphorodiamidate Morpholino Oligomer (CPMO) was evaluated for its antiviral efficacy and cytotoxcity in human cells and neonate murine model. Two CPMOs were designed to block translation initiation of a highly conserved sequence in CHIKV non-structural and structural polyprotein, respectively. Pre-treatment of HeLa cells with CPMO1 signficantly suppressed CHIKV titre, CHIKV E2 protein expression and prevented CHIKV-induced CPE. CPMO1 activity was also CHIKV-specific as shown by the lack of cross-reactivity against SINV or DENV replication. When administered prophylactically in neonate mice, 15 μg/g CPMO1v conferred 100% survival against CHIKV disease. In parallel, these mice demonstrated significant reduction in viremia and viral load in various tissues. Immunohistological examination of skeletal muscles and liver of CPMO1v-treated mice also showed healthy tissue morphology, in contrast to evident manifestation of CHIKV pathogenesis in PBS- or scrambled sCPMO1v-treated groups. Taken together, our findings highlight for the first time that CPMO1v has strong protective effect against CHIKV infection. This warrants future development of morpholino as an alternative antiviral agent to address CHIKV infection in clinical applications.

Chikungunya virus (CHIKV) is an arbovirus primarily transmitted to humans through the bites of the *Aedes aegypti* and *A. albopictus* mosquitoes^1^,^2^. Upon infection with CHIKV, individual succumbs to Chikungunya disease which manifests as sudden-onset fever, severe and persisting arthralgia and/or fatal encelphalitis^1^,^3^,^4^. In recent years, CHIKV has resurged as a significant human pathogen in various tropical and temperate regions worldwide. Since 2005, CHIKV has caused severe unprecedented outbreaks in Indian Ocean islands^5^,^6^,^7^, India^8^,^9^,^10^ and countries in the South-East Asia^11^,^12^,^13^,^14^,^15^. Imported cases of CHIKV infection are increasingly reported in other previously non-endemic areas such as Australia^16^, Europe^17^,^18^,^19^,^20^,^21^ and America^22^,^23^,^24^. Currently, local CHIKV transmission is active in various Carribean countries^23^,^25^. Given its expanding geographical range, CHIKV disease is an emerging key concern to the world public health.

To date, no specific treatment and commercial vaccines for CHIKV infection is available. Symptomatic relief of CHIKV disease through the use of corticosteriods and non-steroidal anti-inflammatory drugs are accompanied with side effects^4^. Broad-spectrum antiviral drugs such as chloroquine and ribavirin are shown effective against CHIKV replication in cultured cells and animal models. However, these drugs showed limited success in human clinical trials^26^,^27^,^28^,^29^. Pharmaco-safety of promising host specific compounds against CHIKV replication have yet to be investigated *in vivo*^26^,^30^, while live attenuated CHIKV vaccines are still in need of further clinical evaluation^31^,^32^,^33^. In this regard, it is of pressing concern for the development of a highly safe and potent therapeutic alternative for CHIKV disease.

CHIKV is an enveloped RNA virus containing a single-strand, positive-sense, 11.8 Kb RNA genome^34^. As with other alphavirues, the viral genomic RNA consists of two open reading frames (ORF). Following receptor-mediated endocytosis of CHIKV into its host cell, the virus mediate mediates synthesis of positive and negative-strand viral RNA and proteins. At the first ORF, CHIKV genomic RNA is translated into a non-structural polyprotein (nsP1–4) which consequently is post-translationally cleaved into non-structural proteins (nsP1, 2, 3 and 4). These non-structural proteins formed a replication complex required to drive the synthesis of a 26S subgenomic RNA. Translation initiation at the second ORF of CHIKV subgenomic RNA leads to formation of structural proteins, namely, Capsid, E1, and E2^34^. Together with newly-replicated viral RNA and structural proteins, mature virions are formed and released. Given that virus replication is dependent on viral RNA synthesis, antisense oligomers targeting specific viral RNA sequence can be effective strategies to inhibit virus replication.

As a recent advance in antisense technology, the Phosphorodiamidate Morpholino oligomer (PMO), is highly efficient in blocking cellular gene expression. PMO is a single-stranded oligonucleotide made up of novel six-membered morpholine ring with purine and pyrimidine bases linked by unique phosphorodiamidate bond^35^,^36^. By high affinity-binding to complementary sequence in the translational region, PMO forms a steric block to ribosome assembly on the target RNA molecule and prevents RNA translation. PMO-mediated inhibition has been widely demonstrated in cell cultures infected with Dengue virus (DENV)^37^, West Nile virus (WNV)^38^, multiple strains of Influenza A virus^39^, Severe Acute Respiratory Syndrome Coronavirus^40^, Enterovirus 71^41^, as well as *Alphavirus* members such as Sindbis virus (SINV) and Venezuelan Equine Encephalitis virus (VEEV)^42^. In murine models, PMO was also strongly protective against WNV,^43^, Influenza A virus^44^, VEEV^42^, Japanese Encephalitis virus^45^,^46^, Respiratory Syncytial virus^47^ and Ebola virus infection^48^. Notably, PMO therapeutic has also successfully reached human clinical trials^49^,^50^.

In prospect of its promising antiviral efficacy, anti-CHIKV PMO, namely, CPMO1 and CPMO2, were designed to target a 25-mer sequence in the AUG region of the first and second ORF of CHIKV RNA genome. This will prevent translation initiation of the non-structural and structural proteins, respectively, and thus inhibit CHIKV replication. Antiviral efficacy was evaluated by viral plaque assays, Western blot detection of viral protein expression, ultrastructural analysis, virus titration and immunohistological analysis of mice organs as well as mice survival study. Our findings collectively showed that CPMO1 was highly potent against CHIKV replication on both cell-based and the murine model for CHIKV.

## Results

### Effective Construct and Efficient Uptake of CPMOs with Absence of Cellular Cytotoxicity

**Figure 1:**
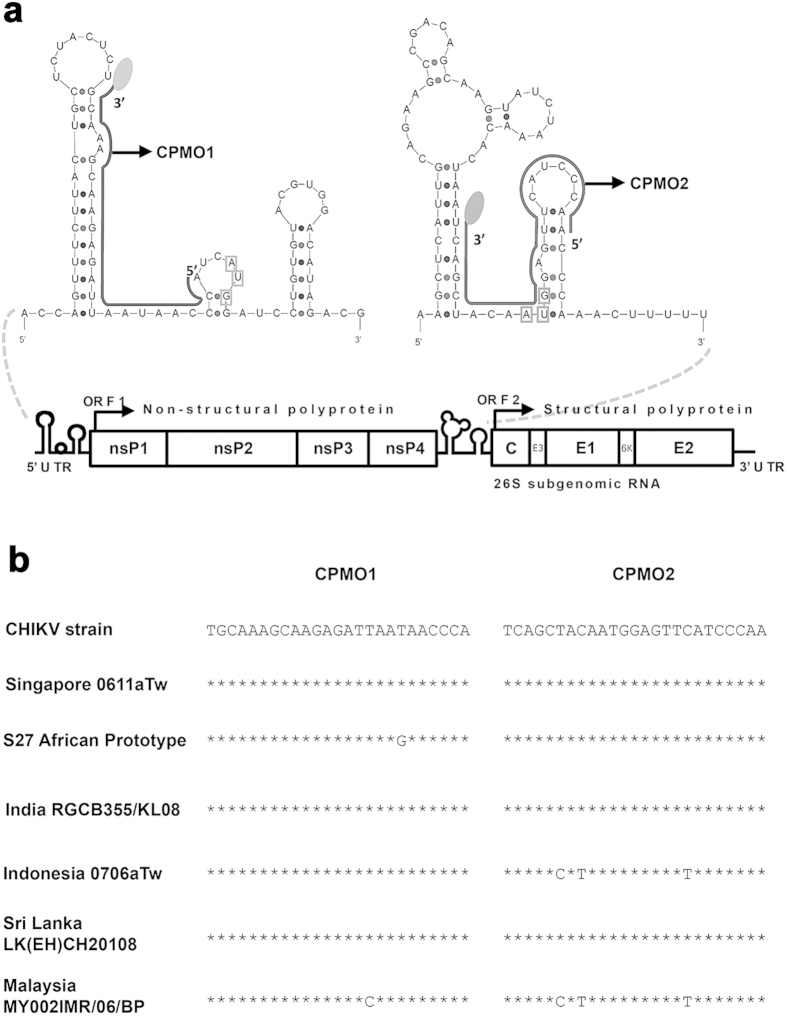
Design of CPMO and scrambled CPMO constructs against CHIKV genomic RNA. (**a**) CPMO1 binds to a sequence (25-mer) upstream of the first Open Reading Frame (ORF), which encodes the non-structural proteins (nsP1–4) while CPMO2 targets a sequence (25-mer) in the second ORF which is important for the expression of Capsid (C), E1 and E2 structural proteins. These structural proteins are required for the formation of the mature CHIKV virion. Black arrows indicate translation initiation. Secondary structures of CPMO target region in the CHIKV genome are predicted by mfold program (http://mfold.rna.albany.edu/?q=mfold/RNA-Folding-Form). Both CPMOs are conjugated to a carboxyflurocesin tag (oval) at the 3’ end. AUG translational start site is outlined in box. CPMO1 target site appears to be more accessible compared to CPMO2. (**b**) NCBI blast nucleotide alignment with representative geographical strains of CHIKV showed high similarity in CPMO target sequences except for a few mismatch base. CPMO; Anti-CHIKV Phosophorodiamidate Morpholino Oligomers. *denotes matched nucleotide base.

**Figure 2:**
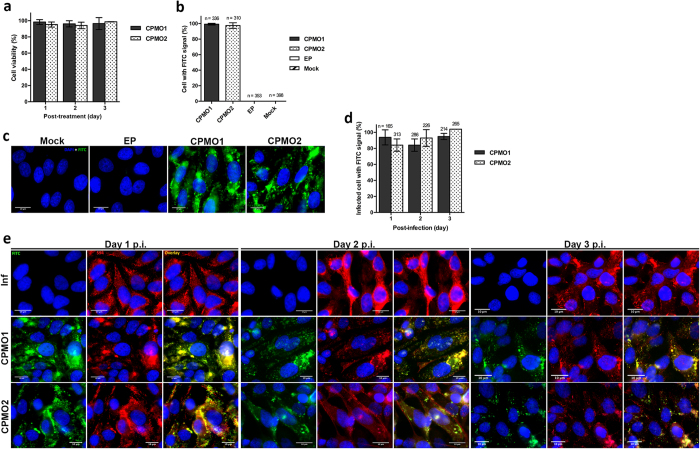
Assessing Cytotoxicity and Efficiency of PMO uptake into HeLa cells. (**a**) CPMO1 and CPMO2 of 10 μM were tested for their cytotoxic effect after day 1, 2 and 3 treatment on confluent HeLa CCL2 cells. At each incubation time point, cell viability was quantitated by AlamarBlue assay and calculated using mock-treated cells as baseline control of 100% viability. (**b**) Cellular uptake of CPMO1 or CPMO2 (10 μM) was quantitated under fluorescence microscopy. HeLa cells were treated with 0 μM (mock-treated) or 6 μM Endo-Porter delivery reagent as vehicle control. Number of cells with intracellular PMO fluorescence signal, n, was indicated on each bar. (**c**) CPMO1 or CPMO2-treated cells were nuclei-stained with dapi dye and observed under DAPI and FITC channels at day 1 post-treatment. (**d**,**e**) Stability of PMO in CHIKV-infected cells was assessed following PMO treatment. Cells pre-treated with CPMO1 or CPMO2 (10 μM) were infected with CHIKV (M.O.I. 0.1), fixed at day 1, 2 and 3 p.i and stained with rabbit anti-CHIKV envelope E2 serum followed by anti-rabbit 594 IgG to assess the stability of PMO in presence of CHIKV infection. (**d**) Cells with positive PMO-FITC signal were expressed as a percentage over the total number of CHIKV-infected cells with E2-594 signal. n number of cells with 594 signal was quantitated and indicated on the top of each bar. Error bars denote the average mean ± s.e.m. expressed from at least two independent set of experiments. (**e**) Representative images under DAPI + FITC, DAPI + TRITC and tri-colours overlay channels were shown under ×100 magnification with scale bar of 10 μm. p.i., post infection; Inf, CHIKV-infected cells; EP, Endo-Porter.

In this study, CPMO1 and CPMO2 were designed to bind to the AUG region of ORF1 and ORF2 of CHIKV genomic RNA, respectively (Fig. 1). Both CPMO target sequences were structurally accessible with no complex secondary folding (Fig. 1a) and the sequences were also predicted to be highly conserved among different geographical strains of CHIKV (Fig. 1b; Table 1). Quantification of cell viability was done following incubation with a combination of PMOs and Endo-Porter (EP) delivery reagent, EP or individual CPMO on confluent HeLa cells for at least 24 h. More than 96% of the cells remained viable across PMO + EP concentration range at 24 h post-treatment (supplementary Figure S1a). Consistently, cell viablity was also close to 100% for EP control treatment (supplementary Figure S1b) and at least 94% was observed for CPMO treatment at 24 h, 48 h and 72 h post-treatment (Fig. 2a). Taken together, this suggests that EP delivery, the designed CPMO and their combination had no apparent toxcity on HeLa cells. In order to visualize CPMO intracellular distribution following cellular uptake, cells were incubated with fluoresceinated-CPMOs (10 μM) and EP (6 μM) for 24 h, stained with DAPI dye and observed under fluorescence microscopy. None of the mock-treated and EP-treated cells have any visible fluorescence signal under FITC channel (Fig. 2b,c). In contrast, 99% of CPMO1 and 97% of CPMO2-treated cells showed diffused fluorescence occuring individually or freely in clusters around the cytosol, thereby suggesting an efficient EP-mediated cellular uptake of CPMO. Next, CPMO-treated cells were subjected to CHIKV infection (M.O.I. 0.1) in an attempt to investigate whether intracellular stability of CPMO is affected during post-CHIKV infection. Fluorescence signals of CPMO1 and CPMO2 were similarly detected in at least 84% of CHIKV-infected cells at day 1, 2 and 3 p.i (Fig. 2d). Similar to post-treatment, CPMO signals were only located in the cell cytosol and most of them colocalized with CHIKV E2 protein stain. This suggests that CPMO could be distributed to the Endoplasmic reticulum where it inhibits CHIKV protein expression. Though CPMO signals were comparatively reduced at day 3 p.i., they have remained intracellularly stable among CHIKV-infected cells as observed by the evident fluorescence signal.

### Significant Reduction of CHIKV titre and viral Protein level in CPMO1-treated Cells

**Figure 3:**
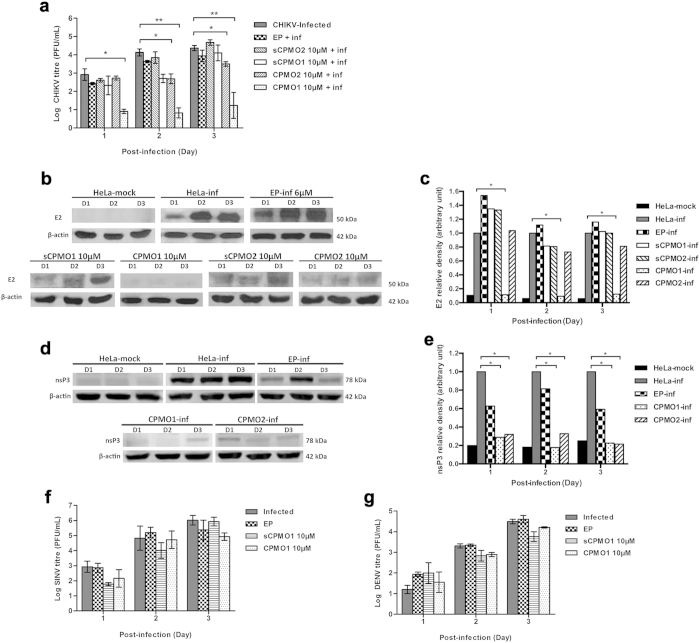
Antiviral efficacy of CPMO1 and CPMO2 in HeLa cells. (**a**) Quantification of CHIKV production from CPMO-treated cells by viral plaque assays. Treatment with Endo-Porter reagent (EP) and scrambled CPMOs (sCPMO1 and sCPMO2) did not interfere with CHIKV replication. CPMO1 treatment (10 μM) produced significant and sustained inhibition against CHIKV replication (M.O.I 0.1) from day 1 (p < 0.01), 2 and 3 p.i (p < 0.001), relative to mock-treated and infected cells. CPMO2 (10 μM) exhibited a lower inhibition relative to the non-treated control. (**b**) Western blot detection of CHIKV E2 protein (50 kDa) expression in total cell lysate of mock-treated, Endo-Porter (EP-inf), scrambled CPMO, or CPMO treated and infected cells across day 1, 2 and 3 p.i. (D1, D2, D3). (**c**) Quantification of relative band density of CHIKV E2 protein. (**d**) Western blot analysis and (**e**) quantification of CHIKV nsP3 protein (78 kDa) level in total cell lysate. β-actin (42 kDa) serves as loading control. Gel image presented was cropped from several original images and all gels were electrophoresed under the same conditions. Treatment of HeLa cells with CPMO1 10 μM did not produce specific inhibition against (**f**) Sindbis and (**g**) Dengue virus replication (M.O.I. 0.1 infection) when compared to the non-treated and scrambled CPMO1 controls. Error bars are indicative of mean ± s.e.m. expressed from three independent set of experiments. Statistical analysis is performed using one-way ANOVA across all groups followed by unpaired one-tailed t-test (*p < 0.05, **p < 0.001).

After pretreatment of HeLa cells with CPMO1 targeting the synthesis of CHIKV non-structural polyprotein or CPMO2 that is targeting the synthesis of structural polyprotein, CHIKV infection was carried out at M.O.I. 0.1 and virus titre in the culture supernatant was quantitated by viral plaque assays. Post-infection time-points, day 1, 2 and 3 were chosen based on CHIKV growth kinetic in HeLa cells, where the highest CHIKV titre was produced at day 3 p.i (supplementary Figure S2). Mock, EP and sCPMOs treatments produced similarly high levels of CHIKV titre from Day 1–3 p.i (Fig. 3a). Relative to mock-treated control, CPMO1 (10 μM) significantly reduced CHIKV titre by 2 log_10_ PFU/ml at day 1 p.i and 3 log_10_ PFU/ml at day 2 and 3 p.i. In contrast, pre-treatment with equal concentration of CPMO2 achieved only a 1 log_10_ PFU/ml reduction in CHIKV titre at day 2 and 3 p.i. In parallel with quantification of virus titre, the total cell lysates of CPMO or sCPMO treated-cells were harvested at all three days p.i for Western blot detection of CHIKV E2 and nsP3 protein expression level. In mock, EP or sCPMO-treated cells, increasing level of expression of CHIKV E2 (Fig. 3b,c) and nsP3 protein (Fig. 3d,e) were observed from day 1 to day 3 p.i, correlating to the increasing virus titres (Fig. 3a). On the other hand, viral E2 and nsP3 protein level was almost completely suppressed at day 1–3 p.i in cells pre-treated with 10 μM of CPMO1. Similarly, CPMO2-treated cells showed strong reduction in both E2 and nsP3 protein expression relative to CHIKV-infected control (Fig. 3b–e). However, CPMO2 treatment seemed less effective in silencing the production of E2 protein and infectious CHIKV titre relative to CPMO1. Taken together, these findings highlight the strong antiviral efficacy of CPMO1 against CHIKV replication.

In the interest of whether CPMO1 is specific in its inhibitory effect to CHIKV per se, we carried out similar treatment assay of CPMO1 on HeLa cells and subjected them to Sindbis virus (SINV) or Dengue virus (DENV) infection at M.O.I 0.1 for day 1–3 p.i. SINV was used in this study as it belongs to the *Togaviridae* family similar to CHIKV while DENV is a representative member of the flaviviruses. Quantification of virus titre by viral plaque assays demonstrated no significant difference in SINV or DENV titre for CPMO1-treatment relative to the mock-treated cells (Fig. 3f,g). In support of this data, homology alignment of CPMO1 target sequence in CHIKV RNA (GenBank; FJ445502) to SINV (GenBank; NC_001547) and DENV genome (GenBank; M29095.1) revealed no significant similarity (data not shown). Therefore, we deduce that CPMO1 is highly specific against CHIKV with no cross-reactivity to the replication of other alphaviruses or flaviviruses.

### Ultrastructural Analysis of CPMO1-treated Cells Reveals Absence of CHIKV Replication

**Figure 4:**
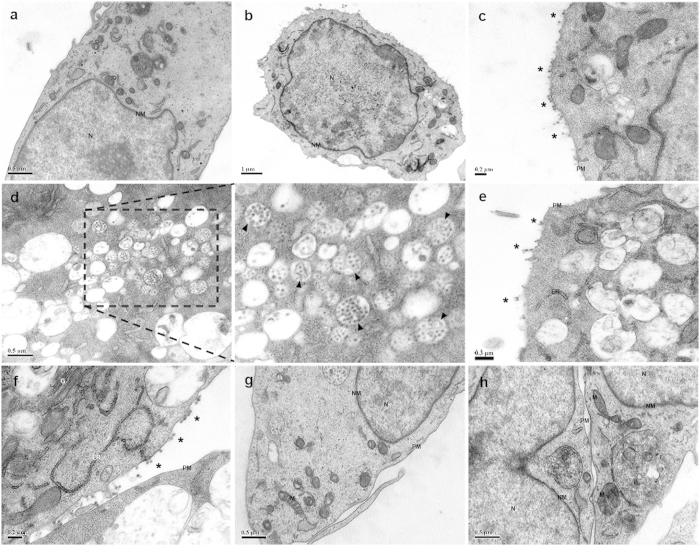
Transmission electron microscopy is performed at day 3 p.i. (M.O.I. 0.1) of (**a**) mock-treated and mock-infected, (**b**–**d**) mock**-**treated and CHIKV-infected, (**e**) treated with 6 μM Endo-Porter, (**f**) treated with 10 μM sCPMO1 and (**g**,**h**) 10 μM CPMO1. (**d**) CHIKV-induced cytopathic vacuoles, CPV II, are indicated by arrowheads and virus budding from cell surface membrane is indicated by *. Representative images are shown here with corresponding scale bar in μm. CPV II, cytopathic vacuole II; ER, endoplasmic reticulum; G, Golgi apparatus; L, lysosome; M, mitochondria; N, nucleus; NM, nuclear membrane; PM, plasma membrane.

To further validate the efficacy of CPMO1, transmission electron microscopy (TEM) was carried out on day 3 post-infected cells for ultrastructural observation of CHIKV replication. Mock-infected HeLa cells maintained healthy cellular morphology with intact organelles such as a well-defined nucleus, mitochondria and endoplasmic reticulum (Fig. 4a). The cell surface membrane was smooth and there was no sign of CHIKV virions. In CHIKV-infected (Fig. 4b–d), EP- (Fig. 4e) and sCPMO1-treated cells (Fig. 4f), extensive virus replication has occurred as indicated by the formation of numerous CHIKV replication complexes, namely, the cytopathic vacuoles type II (CPV-II, arrowheads) in the cytosol (Fig. 4d). During the late phase of CHIKV replication, infected cells showed assembly and budding of mature CHIKV virions from cell surface and intercellular junctions (Fig. 4c,e,f). In contrast, CPMO1-treated cells showed no sign of CHIKV replication with the absence of CPVs at say 3 p.i. (Fig. 4g,h). The mitochondria and endoplasmic reticulum remained intact and there was no virus budding from cell surface and in between cell junctions, similar to the mock-treated cells (Fig. 4a). These ultrastructural analysis further strengthen our finding that treatment of HeLa cells with CPMO1 has effectively prevented CHIKV infection within the cells.

### Strong Protection of CPMO1v against CHIKV Infection in Mice

**Figure 5:**
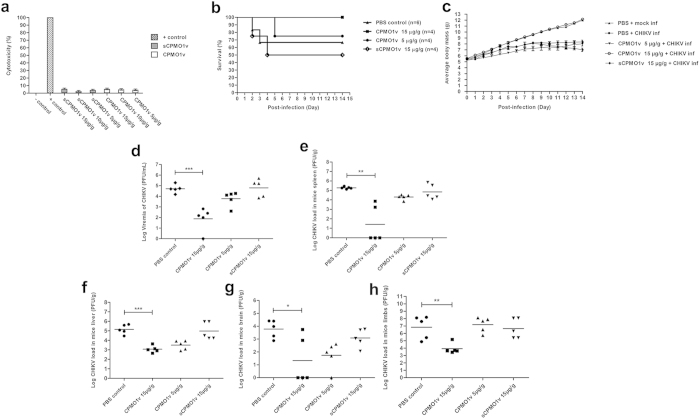
Evaluation of *in vivo* toxicity and anti-CHIKV efficacy of CPMO1v in neonate murine model. (**a**) BALB/c mice (6-day old, n = 6 per group) were intraperitoneally (i.p) injected with sterile PBS, anti-CHIKV vivo-PMO (CPMO1v) or scrambled vivo-PMO (sCPMO1v) at 5 μg/g, 10 μg/g or 15 μg/g consecutively for seven days at every 24 h interval. Mice were sacrificed at 6 h after the last treatment and whole blood was harvested for quantification of LDH activity level. (**b**) In survival study, two dose of sterile PBS, CPMO1v or sCPMO1v at 5 μg/g or 15 μg/g were given via i.p to neonate mice (n = 6 per group) at 24 h interval, followed by CHIKV infection at 4 × 10^5^ PFU. Treatment was given at two more equivalent doses at 18 h p.i. and 42 h p.i. and mice were monitored daily for sign or symptom of CHIKV morbidity as well as (**c**) their daily weight gain over two weeks. At the end of two weeks p.i., the number of surviving mice was recorded and presented on Kaplan-Meier chart. A dose-dependent protection is observed in CPMO1v-treated mice relative to the PBS- and sCPMO1v- control groups. (**d–h**) Mice (n = 5 per group) were subjected to the same treatment regimen above. At 48 h p.i., (**d**) whole blood, (**e**) spleen, (**f**) liver, (**g**) brain and (**h**) hind limbs were harvested and homogenized for viral plaque assays. Significant reduction in viremia and CHIKV load were observed. Statistical analysis is done using one-way ANOVA across all groups followed by unpaired one-tailed t-test. (*p < 0.05, **p < 0.005, ***p < 0.0005; Graphpad prism 6).

Having shown that CPMO1 could strongly suppress cellular CHIKV replication, we further assessed the antiviral efficacy of CPMO1v in suitable murine model (6-day old BALB/c mice) that was previously established for CHIKV infection in our research laboratory. In this 6-day old BALB/c model, mice succumbed to CHIKV infection with a range of 10–65% lethality in day 5–9 p.i. relative to mock-infected wildtype control group. There could be variability in survival due to the development of innate immunity that help mice to overcome lethality. Therefore, it is more notable for the use of this mouse model to analyze the acute phase of CHIKV infection, where the latter was well-characterized by high viremia, virus burden and pathogenesis in CHIKV-targeted tissues including the hind limb muscles, liver, spleen and brain (data not shown). In this study, Vivo-PMOs, namely, CPMO1v and scrambled CPMO1v (sCPMO1v), were synthesized where their fluorescence dye was replaced with an octaguanidium dendrimer to mediate effective *in vivo* delivery to mice tissues. Following CPMO1v or sCPMO1v administration via intraperitoneal (i.p) injection at 5, 10 and 15 μg/g to our murine model, serum Lactate dehydragenase (LDH) level in the mice remained low and insignificant compared to the LDH positive control. Activity of LDH is commonly used as a stable biomarker in cytotoxicity assay^51^,^52^. Based on this finding, we inferred an absence of dose-dependent toxicity from vivo-PMO treatment (Fig. 5a). Next, the antiviral efficacy of CPMO1v at 5 μg/g or 15 μg/g was investigated where the compound was administered to the mice for two consecutive days, followed by CHIKV infection and subsequent treatment with CPMO1v for two more consecutive dose at 18 h and 42 h p.i. A dose-inhibition effect was observed where 5 μg/g of CPMO1v protected 75% of the mice from CHIKV-induced morbidity and 15 μg/g has further enhanced the survival of all mice for two weeks p.i. (Fig. 5b). In contrast, PBS- and sCPMO1v control groups showed 65% survival at Day 3 p.i. and 50% survival at Day 4 p.i, respectively. On average daily basis, 15 μg/g CPMO1v-treatment also conferred the mice healthy weight gain similar to mock-infected mice while 5 μg/g CPMO1v-treated mice showed retarded growth similar to PBS- and sCPMO1v-treatment groups (Fig. 5c). As this mouse model did not provide evaluation of absolute lethality, quantification of CHIKV titre in mice serum and several organs was further evaluated at day 2 p.i. to analyze the protective efficacy of CPMO1v during the acute phase of CHIKV infection. Indeed, strong suppression of CHIKV production in various tissues of CPMO1v-treated mice relative to PBS- and sCPMO1v-treated groups (Fig. 5d–h). In line with high potency of protection seen in survival study, CPMO1v given at 15 μg/g had the most notable reduction of viremia (2.83 log_10_; Fig. 5d), CHIKV load in the spleen (3.85 log_10_; Fig. 5e), liver (2.09 log_10_; Fig. 5f), brain (2.45 log_10_; Fig. 5g) and limbs (2.91 log_10_; Fig. 5h). Taken together, these data showed that the administration of 15 μg/g CPMO1v strongly protects against CHIKV disease in our murine model during the acute phase.

### Absence of CHIKV Pathology in Tissues of Mice pre-treated with CPMO1v

**Figure 6:**
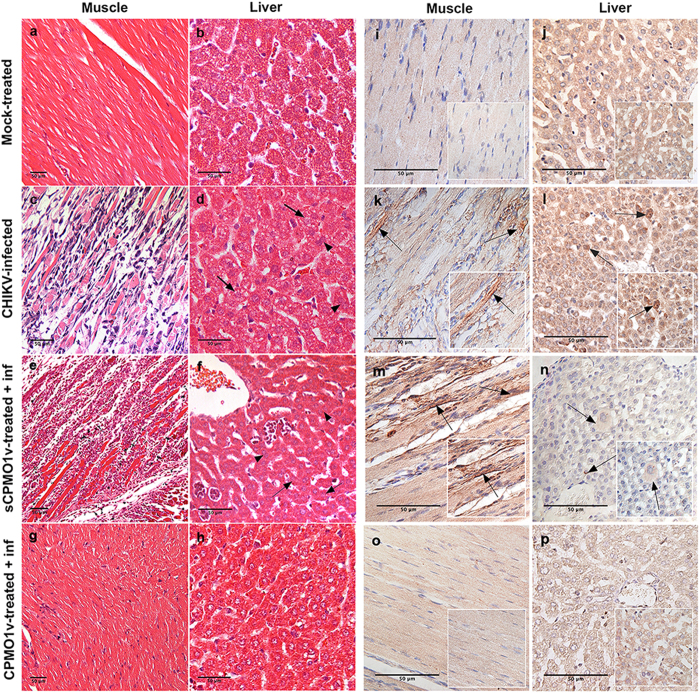
Histological analysis of mice pre-treated with CPMO1v. BALB/c mice (6-day old, n = 6) were i.p injected with 15 μg/g CPMO1v or sCPMO1v. CHIKV infection and treatment regimen is carried out as aforementioned in Fig. 5. All mice were sacrificed at day 7 p.i. and their hind limbs and liver were harvested, fixed with formalin, ethanol-dehydrated and paraffin embedded in sections for (**a–h**) H&E staining or (**i–p**) IHC staining using primary rabbit anti-CHIKV E2 IgG followed by secondary goat anti-rabbit HRP IgG. (**d**) **&** (**f**) Arrow indicates smudgy nuclei inclusions while arrowhead indicates multi-nucleated inclusions of pale necrotic hepatocytes. (**k**,**l**) Arrow indicates positive CHIKV antigen staining. Images were viewed and captured at 400× under Olympus microscope. Representative images at 50 μm scale are shown.

To further validate the antiviral activity of CPMO1v against CHIKV disease development in mice, histological analysis was performed on PBS-, sCPMO1v- and CPMO1v-treated mice tissues at day 7 p.i. PBS-treated and CHIKV infected mice tissues showed evident morphological abnormities. In line with studies on CHIKV muscle tropism^50^,^53^,^54^,^55^, limb muscle fibres of PBS- or sCPMO1v-treated and CHIKV-infected mice showed degenerative changes in the form of diffused necrosis and extensive infiltration of various immune cells such as lymphocytes and neutrophils (Fig. 6c,e). This suggests that inflammation has occurred in the skeletal muscle fibres due to CHIKV pathogenesis. Liver tissues of these mice also showed nuclei inclusions of pale necrotic hepatocytes indicative of CHIKV-induced morphological damage (Fig. 6d,f). In contrast, muscle and liver tissues of 15 μg/g CPMO1v-treated mice retained healthy integrity and were protected from CHIKV pathogenesis (Fig. 6g,h), similar to mock-treated mice (Fig. 6a,b), respectively.

In parallel with findings from H&E examination, immunohistochemical (IHC) analysis showed positive and intense CHIKV antigen staining in the limb muscle and liver of PBS-treated mice at day 7 p.i. In both PBS or sCPMO1v-treated groups, CHIKV antigens were widely distributed along the epimysium of the skeletal muscle (Fig. 6k,m) and viral antigenic staining appeared like granule structures in the cytoplasm of hepatocytes (Fig. 6l,n). This is closely associated with the degeneration and necrosis of hepatocytes shown in H&E staining (Fig. 6d,f). In contrast, CPMO1v-treated mice were strongly protected with no CHIKV antigens in both muscle and liver tissues (Fig. 6o,p). In summary, these histological examinations visually validate our findings thus far that CPMO1v is strongly protective against CHIKV replication in mice following CHIKV infection.

## Discussion

In this study, PMO was investigated for its inhibitory efficacy against CHIKV replication in human cells and in the murine model. Our previous study, which utilized a small hairpin RNA (shRNA) construct, demonstrated the strong potential of antisense molecules in suppressing CHIKV replication in HeLa cells^56^. However, treatment-induced toxicity and the high susceptibility of siRNA to intracellular degradation posed as limitations in furthering the development of shRNA for pre-clinical applications^57^,^58^. In this regard, an improved antisense technology with desirable biochemical characteristics is required. PMOs are neutral DNA-like oligomers made up of non-ionic phosphorodiamidate backbone. This unique construct allows PMO to possess high cellular stability and excellent safety profile^36^,^59^. In this study, we have designed two PMOs, CPMO1 and CPMO2, that targets CHIKV RNA genome and evaluated them for their toxicity and inhibitory effect against CHIKV replication both *in vitro* and *in vivo*. We found that CPMOs exert no cytotoxic effect on HeLa cells and intraperitoneal (i.p.) administration of the highest dose 15 μg/g CPMO1v or sCPMO1v to our established murine model (6-day old neonate BALB/c mouse) also showed an absence of PMO-induced toxicity and lack of behavioural abnormality. Similarly, safe pharmacological profile of PMO in cell cultures and mice have also been observed in other PMO studies on flaviviruses replication^45^,^60^. To enhance *in vivo* delivery, CPMO1v evaluated in our neonate murine model was conjugated to an octaguanidinium dendrimer. This arginine-rich peptide has been well-validated for excellent systemic delivery of vivo-morpholino into a wide-array of mice tissues, albeit a reduced penetrating ability into the brain due to the low permeability of the blood-brain barrier^61^,^62^. Here, we observed significant reduction of CHIKV load in the liver, spleen, skeletal muscles as well as the brain following i.p treatment of CPMO1v. This suggests CPMO1v could have been favorably taken up into these organs and therefore prophylactically protecting the tissues against CHIKV replication. To strengthen this speculation, further investigation on CPMO1v biodistribution using bioanalytical tools^63^ can be carried out.

Based on previous studies, HeLa cells are well-established to be highly permissive to CHIKV entry and replication^56^,^64^,^65^. Thus in this study, HeLa cells were appropriated as the human cell model to address the efficacy of CPMOs against CHIKV replication. In addition, the CPMOs could have broad-spectrum activity as each of them targets a highly conserved AUG region sequence among CHIKV strains of different lineages. Furthermore, CPMO1 was CHIKV-specific as it did not cross-react with SINV and DENV replication. Strong efficacy of CPMO1 in suppressing CHIKV replication in HeLa cells was demonstrated collectively in viral plaque assays, Western blot analysis and ultrastructural analysis. In contrast, CPMO2 only caused a reduction of 1 log_10_ PFU/ml in CHIKV titre (Fig. 3a,b). This could be attributed to the difference in CPMO target sequence. During the early stage of CHIKV replication, the non-structural proteins (nsP1–4) form a viral replication complex that mediates the synthesis of a full-length negative-strand RNA^34^. At late stage of CHIKV replication, the negative-strand RNA then directs the synthesis of positive-strand genomic RNA required for new virions formation and production of structural proteins. Here, we postulate that CPMO1 could have a dual blocking effect, first, on the synthesis of CHIKV non-structural viral polyprotein, followed by a secondary inhibition on downstream positive-strand RNA synthesis. As CPMO1 targets upstream of the AUG codon in the first ORF, it blocks the translation of CHIKV genomic RNA to non-structural viral polyprotein. Indeed, our findings indicate significant reduction in CHIKV titre and knockdown of CHIKV E2 protein synthesis sustained from day 1–3 p.i, suggesting that CPMO1 suppresses non-structural protein synthesis at early stage of CHIKV replication (prior to day 1 p.i.) and this consequently reduced CHIKV structural protein synthesis and infectious virions production at late stage of CHIKV replication (day 2–3 p.i.). On the other hand, CPMO2 targets the AUG region in the second ORF and this hinders the translation of CHIKV subgenomic RNA to structural proteins, namely, the Capsid, E1 and E2. Therefore, CPMO2 is likely to exhibit its inhibitory activity at the late stage of CHIKV replication. Indeed, significant CPMO2-mediated reduction in CHIKV titre was observed only at day 2 and 3 p.i. and this was accompanied with a moderate decrease in E2 protein production at day 1–3 p.i. At these experimental time-points, negative sense RNA production could have occurred extensively and CPMO2 may not be sufficiently effective to silence all structural protein synthesis. Consequently, the inhibition was seen lower than that achieved by CPMO1, which can target an early step of CHIKV replication. Based on this interpretation, we can also deduce that CPMO is sequence-dependent for its antiviral activity. The control sCPMOs have neither caused non-specific reduction in CHIKV titre nor drastic decrease in E2 protein level. This signifies that for future studies requiring specific silencing of a stage of CHIKV replication, more CPMO candidates can be similarly designed and screened.

In order to confirm the strong cellular anti-CHIKV efficacy of CPMO1, we established a treatment regimen in CHIKV-infected murine model. Neonate mice were pre-treated with CPMO1v followed by two additional dose after CHIKV challenge. Survival study demonstrated that 15 μg/g CPMO1v conferred 100% survival and normal daily weight gain over 14 days p.i. In contrast, PBS- and sCPMO1v-treated mice were susceptible to CHIKV disease where these mice had poor weight gain, paralysis of single hind limb on day 2 p.i. and their mortality was significantly reduced to 65% and 50%, respectively, at the end of two weeks. Other murine studies have reported that CHIKV primarily infects the liver, muscles, joints, spleen and brain, displaying a tropism characteristic of CHIKV pathogenesis in human^53^,^54^,^55^. Thus in our murine model, quantification of viremia, virus load and examining histopathologic changes are necessary to further investigate the antiviral efficacy of CPMO1v. Here, we found that viremia was significantly reduced by 2.8 log in mice treated with 15 μg/g CPMO1v as well as at least 2 log reduction of virus load in the spleen, liver, brain and limbs, collectively. Skeletal muscles of the hind limbs and liver of CPMO1v-treated mice displayed healthy tissue morphology with no sign of CHIKV-induced degenerative damage (Fig.6g,h,o,p). Comparatively, gross histopathologic changes and strong signal of CHIKV antigen distribution were observed in the muscle fibres and liver tissue of PBS- and sCPMO1v-treated mice, as consistent with other CHIKV murine models established^53^,^54^,^55^,^66^. In summary, these findings provide compelling evidence on the strong efficacy of CPMO1v against CHIKV replication. We postulate that the specific translational-blocking activity of CPMO1v on CHIKV non-structural and structural protein synthesis has exerted a histological protection in mice against the onset of CHIKV pathogenesis.

In conclusion, this is a novel study demonstrating the strong prophylactic efficacy of morpholino oligomer in inhibiting CHIKV replication on cell-based and murine models. With these findings, it may be necessary to evaluate the robustness of CPMO1 against resistant virus mutant generation as well as the therapeutic efficacy of CPMO1 administration after CHIKV challenge. Nevertheless, our study revealed a proof-of-concept on the antiviral efficacy and sequence-dependent specificity of morpholino against CHIKV replication. In the long run, further evaluation is required to develop morpholino into a translational antiviral therapy for CHIKV disease.

## Methods

### Cells and Viruses

HeLa (ATCC No. CCL-2), BHK (ATCC No. CCL-10) and C6/36 (ATCC No. CRL-1660) cells were used in this study. HeLa cells were subcultured in DMEM medium while BHK-21 cells were subcultured in RPMI-1640 medium. Both HeLa and BHK cells were incubated at 37 °C in 5% CO_2_. C6/36 mosquito cells were maintained in L-15 medium at 28 °C. All culture media were supplemented with 10% heat-inactivated FCS and cells were maintained in T75 cm^2^ tissue culture flask.

CHIKV used in cellular infection studies was SGEHICHD122508 (GenBank; FJ445502), a virus isolate from the serum of an infected patient in Singapore and was kindly provided by Environmental Health Institute, National Environmental Agency (Singapore). CHIKV LK(EH)CH6708 (GenBank; FJ513654) was used in mice studies. To establish an infectious CHIKV pool, confluent C6/36 monolayer in T75 cm^2^ flasks was infected with CHIKV and cells were maintained in L-15 media with 2% FCS for 3–4 days post-infection (p.i.). Supernatants were harvested and CHIKV titre was determined by standard viral plaque assays. Similarly, DENV NGC-2 strain (GenBank; M29095.1) and SINV (GenBank; NC_001547) were propagated in C6/36 cells and BHK cells, respectively.

### Design of CPMO

CPMO1 and CPMO2 (GeneTools, USA) were designed to be complementary to a 25-mer sequence located in the AUG region (ORF1 and ORF2) of the CHIKV genomic RNA (SGEHICHD122508 virus isolate, GenBank; FJ445502) (Fig. 1a; Table 1). Scrambled morpholinos, sCPMO1 (1^st^ BASE, Singapore) and sCPMO2 (GeneTools, USA) with random base sequence of their respective CPMO were synthesized as negative controls. To visualize cellular uptake and cytosolic distribution, all CPMOs and sCPMOs were covalently conjugated at 3’ end with carboxyfluorescein. For mice studies, the Vivo-morpholino (GeneTools, USA) named as CPMO1v and scrambled CPMO1v (sCPMO1v), were synthesized with an octa-guanidine dendrimer for facilitating *in vivo* tissue uptake of the oligomer. Using NCBI BLASTn tool, CPMOs were aligned to all published CHIKV genomic sequences (CHIKV taxid: 37124), with representative geographical isolates such as the S27-African prototype of the Central/East African (CEA) genotype (GenBank; AF369024), India RGCB355/KL08 (GenBank; GQ428214), Indonesia 0706aTw (GenBank; FJ807897), Singapore 0611aTw (GenBank; FJ807896), Sri Lanka LK(EH)CH20108 (GenBank; FJ513679) of the ECSA genotype, and Malaysia MY002IMR/06/BP (GenBank; EU703759) of the Asian genotype shown in Fig. 1b. Comparative sequence alignment of CPMO1 to SINV (GenBank; NC_001547) and DENV (GenBank; M29095.1) were also performed (data not shown).

### Cell Viability Assay

HeLa cells were seeded at 90% confluency on 96-well plate and 24 h later, Endo-Porter cellular delivery reagent at 0 μM, 2 μM, 4 μM, 6 μM and 8 μM, CPMO at 10 μM or a combination of Endo-Porter at 6 μM and CPMO or sCPMO at 10 μM was added to the culture media. At day 1, 2 and 3 post-treatment, alamarBlue cell viability test was carried out following the manufacturer’s protocol. The resultant fluorescence was read by Infinite™ 200 series microplate reader (Tecan i-control, Switzerland). Cell viability was expressed as a percentage using 100% viability of the mock-treated HeLa cells as baseline negative control. At least two independent experiments were performed in triplicates.

### Pre-treatment of Cells with CPMOs & Assessing Cellular Uptake using Immunofluorescence Assay

Confluent monolayer of HeLa cells on 24-well plates were treated with 10 μM of CPMO1 or CPMO2. CPMO was pre-mixed with 6 μM of Endo-Porter reagent before adding to the cells and at least two independent treatments, each in triplicates, were carried out. At day 1 post-treatment, HeLa cells were washed twice with PBS, fixed with 4% paraformaldehyde in PBS and permealized with 0.1% Triton-X. For analysis of CPMO distribution in CHIKV-infected cells, CPMO-treated cells were infected with CHIKV (MOI 0.1) at day 1 post-treatment. Cells were fixed at day 1, 2 and 3 post-infection, fixed and permeabilized as described. Cells were incubated with rabbit anti-CHIKV E2 13893 B3 polyclonal antibody (in-house produced) at 1:100 dilution followed by secondary goat anti-rabbit DyLight 594 at 1:300 dilution (Thermoscientific, USA). Antibody incubation was performed for 1 h each and dilution was performed in PBS. Finally, cell nuclei were stained with DAPI (Duolink *in situ* mounting media; Sigma-aldrich), viewed and quantitated for positive signal under DAPI, FITC and TritC channel at 100× magnification (Olympus IX81 inverted microscope).

### Virus Infection Assay

HeLa cells pretreated with CPMO or sCPMO were infected with CHIKV, SINV or DENV at M.O.I. 0.1. During 1.5 h virus infection, cells were incubated at 37 °C in 5% CO_2_ with rocking of the plates at every 15 min. Thereafter, excess virus was washed off with PBS and cells were maintained in DMEM supplemented with 2% FCS at 37 °C in 5% CO_2_. Culture supernatants were harvested at Day 1, 2 and 3 p.i. At these time points, total cell lysates of CHIKV infected cells were also collected by scraping cells in ice cold M-PER reagent containing Halt Protease inhibitor cocktail and 1% EDTA solution (Thermoscientific).

### Viral Plaque Assay

BHK cells were seeded at 90% confluency on 24-well plates for quantification of the infectious virus titre in the harvested culture supernatants, mice serum or homogenized organs. At the end of infection, cells were maintained in an overlay media (1% Carboxymethyl cellulose and RPMI-1640 media supplemented with 2% FCS) and plates were incubated at 37 °C in 5% CO_2_. At three days p.i. of CHIKV infection, cells were stained with crystal violet dye. For SINV or DENV titration, cells were stained with crystal violet dye at two days p.i. or seven days p.i., respectively. Virus titre was then quantitated by the number of plaques formed in the stained monolayer.

### Western blot Analysis

Total cell lysates harvested at day 1, 2 and 3 p.i. were quantitated by BCA assay (Thermoscientific) and 25 μg of each lysate was subjected to SDS-PAGE. CHIKV E2 protein was probed using rabbit anti-CHIKV E2 13893 B3 polyclonal antibody (in-house produced) at 1:3000 dilution and goat anti-rabbit secondary antibody conjugated to HRP (Thermoscientific) at 1:6000 dilution. On the other hand, CHIKV nsP3 protein was probed using rabbit anti-nsP3 antibody (in-house produced) at 1:100 dilution followed by secondary anti-rabbit HRP antibody at 1:10,000 dilution. Beta-actin detection was carried out using mouse monoclonal anti-actin at 1:6000 or 1:10,000 (Millipore, USA). CHIKV E2 blot was probed with antibodies in TBST with 5% skim milk while CHIKV nsP3 blot was probed with PBST with 5% BSA. Blots were washed using TBS or PBS buffer with 0.1% Tween-20. Following incubation with antibodies, protein bands were developed by Enhanced Chemiluminescence (ECL) method using Supersignal® West Pico Chemiluminescent Substrate (Thermoscientific). Quantification of relative band density was performed using Image J^67^, followed by one-way ANOVA analysis with reference to CHIKV-infected control bands.

### Transmission Electron Microscopy

HeLa cells were seeded at 90% confluency on 6-well plates and treated with CPMO1 or sCPMO1 at 10 μM, Endo-Porter or mock-treated, respectively. At 24 h post-treatment, cells were washed with PBS and infected with CHIKV at M.O.I. 0.1. At day 3 p.i., cells were fixed and processed for TEM as described in our laboratory-established protocol^56^.

### Ethics statement

All mice experiments were approved and performed in accordance to the guidelines provided by Institutional Animal Care and Use Committee of National University of Singapore (Protocol No. 023/12).

### Mice and CPMO1v Treatment

Neonate BALB/c mice were used in this study. The animals were housed in a pathogen-free BSL2-facility in Vivarium, CeLS building in National University of Singapore. Six-day-old mice (n = 5 or 6 per group) were treated with 5 μg/g or 15 μg/g of CPMO1v, sCPMO1v diluted in sterile PBS or mock-treated with sterile PBS only by intraperitoneal (i.p) injection for two consecutive dose at every 24 h interval. At 6 h following the second dose, mice were i.p injected with 10^5^ PFU of CHIKV (strain LK (EH) CH6708, GenBank; FJ513654). At 18 h and 42 h p.i, equivalent dose of vivo-PMO or PBS was given. At the end-point of 48 h p.i., mice were sacrificed under anesthesia and whole blood, brain, spleen, liver and limbs were harvested for quantification of CHIKV titre. Tissues were homogenized using Precellys CK beads and centrifuged at 14,000 g × 10 min to obtain clarified supernatant for viral plaque assay. For survival analysis, mice (n = 6) were pretreated as described above. Following CHIKV infection, mice were monitored daily and scored for sign and symptoms of CHIKV disease. At the end of two weeks, the number of surviving mice was noted and all mice were humanely euthanized and sacrificed.

### Toxicity Evaluation of Vivo-PMO in Mice

BALB/c mice (6-day old, n = 6) were i.p injected with sterile PBS, CPMO1v or sCPMO1v at 5, 10 or 15 μg/g at every 24 h for 7 days. At 6 h after the last treatment, mice were sacrificed and whole blood was collected for evaluation of LDH level following the manufacturer’s protocol in the LDH Cytotoxicity Assay kit (Biovision, USA).

### Histological and Immunohistochemical Examination of Infected Mice tissues

BALB/c mice (6-day old, n = 3) were pretreated with sterile PBS, CPMO1v or sCPMO1v and subjected to CHIKV infection as described above. At day 7 p.i., mice were sacrificed and their limbs and liver were harvested and formalin fixed, dehydrated and paraffin embedded. Sectioning of tissue samples was done at 4 μm and samples were routinely stained with haematoxylin and eosin (H&E). For IHC, tissue samples were labeled with primary rabbit E2 antibody diluted to 1:100, followed by a secondary goat anti-rabbit HRP conjugate (Thermoscientific). The assay was subsequently performed using automated Bond-Max System (Leica Biosystems, Germany) based on a customized protocol with several steps of dehydration and differentiation. Final slides were then mounted with PermountTM mounting medium (Fisher Chemical, UK) and viewed under Olympus microscope. Images were captured at 400× magnification to visualize CHIKV antigen.

## Additional Information

**How to cite this article**: Lam, S. *et al*. Antiviral Phosphorodiamidate Morpholino Oligomers are Protective against Chikungunya Virus Infection on Cell-based and Murine Models. *Sci. Rep*. **5**, 12727; doi: 10.1038/srep12727 (2015).

## Supplementary Material

Supplementary Information

## Figures and Tables

**Table 1:** Antisense CPMOs and scrambled CPMO sequences (25-mer).

Construct	Sequence (5’ – 3’)^a^	Sequence Homology (%)^b^	Target site in CHIKV genome
CPMO1/CPMO1v	TGGGTTATTAATCTCTTGCTTTGCA	56.1	26–50
sCPMO1/sCPMO1v	**GT**G**TA**T**TGATTATC**C**C**T**TTCGATGT**		—
CPMO2	TTGGGATGAACTCCATTGTAGCTGA	62.1	7531–7570
sCPMO2	**G**T**T**G**AG**TGAACT**A**C**C**T**GT**T**GA**C**GT**A		—

^a^The mispaired bases of the scrambled CPMO (sCPMO) sequences relative to CPMO are presented in bold and underlined.

^b^CPMO1 and CPMO2 sequence are 100% conserved in query coverage and identity to 56.1% and 62.1% of all published CHIKV strains in NCBI database.
