# Supplementary material for: Antiviral Phosphorodiamidate Morpholino Oligomers are Protective against Chikungunya Virus Infection on Cell-based and Murine Models
Source: Sci Rep. 2015 Jul 30;5:12727. doi: 10.1038/srep12727 (PMC4649900; doi:10.1038/srep12727)
Supplement: Supplementary Information [file srep12727-s1.doc]

**Antiviral Phosphorodiamidate Morpholino Oligomers are Protective against Chikungunya Virus Infection on Cell-based and Murine Models**

Shirley Lam, Huixin Chen, Caiyun Karen Chen, Nyo Min and

Justin Jang Hann Chu*

**Supplementary Figure S1**

**a**


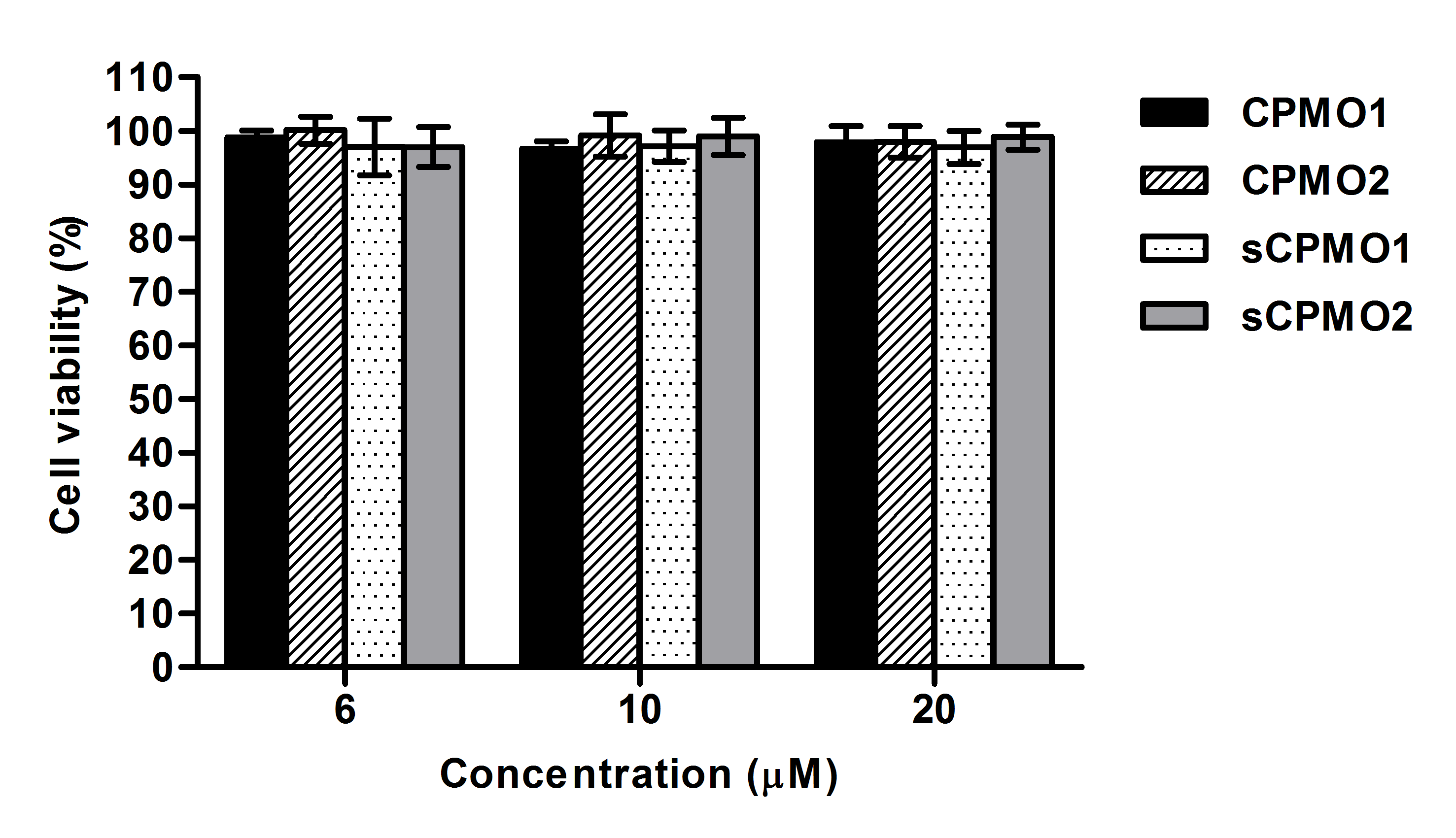

**b**


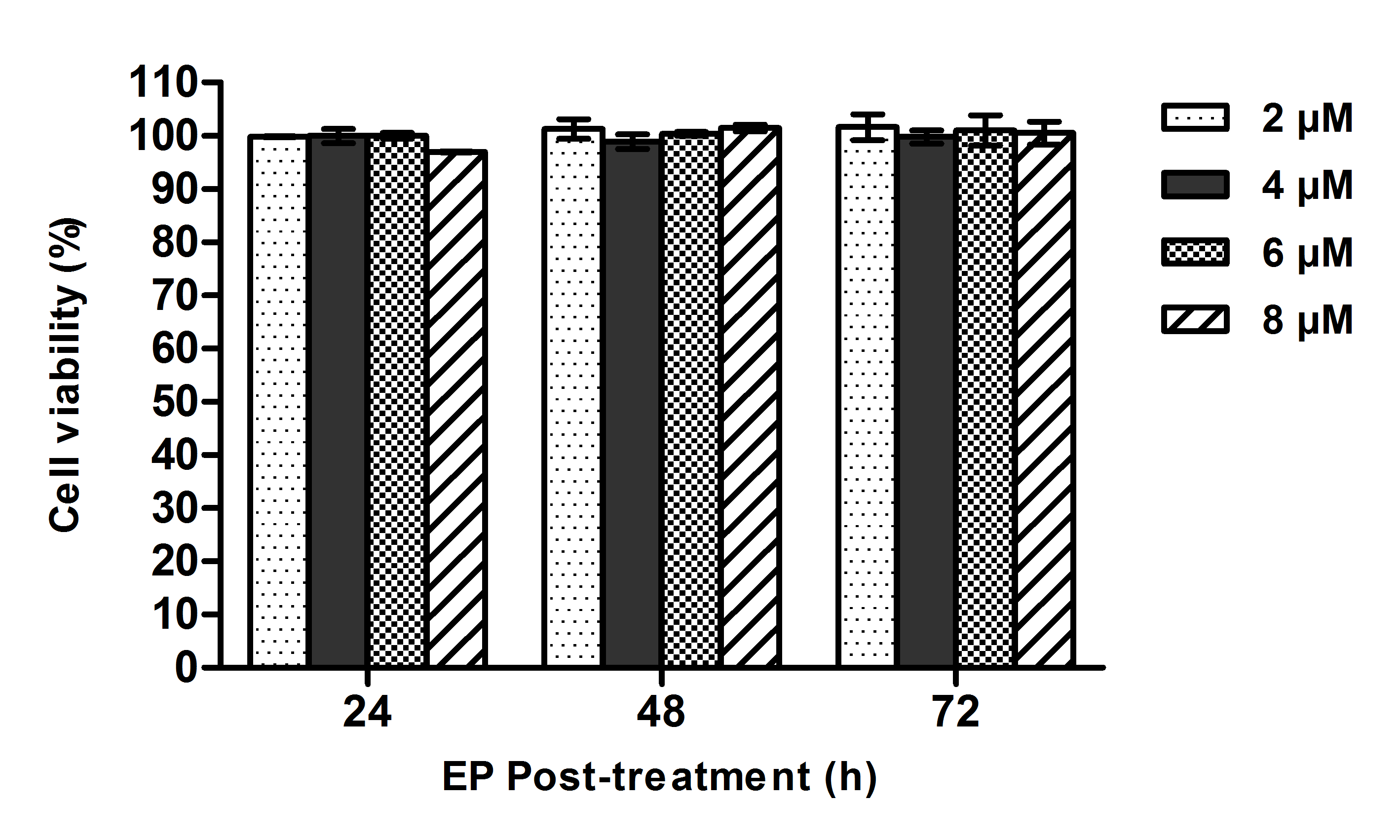


**Figure S1 (a)** A range of CPMO and scrambled CPMO (sCPMO) of 6, 10 and 20 µM were tested for their cytotoxic effects after 24 h treatment on HeLa cells. **(b)** Endo-Porter (EP) delivery reagent of 2, 4, 6, 8 µM was tested at 24 h, 48 h and 72 h pre-treatment on confluent HeLa CCL2 cell monolayer. After each incubation time point, cytotoxic effect was quantitated using AlamarBlue assay. Percentage cell viability is calculated using mock-treated cells as baseline control of 100% viability. Error bars denote the average mean ± s.e.m. expressed from two independent set of experiments.

**Supplementary Figure S2**


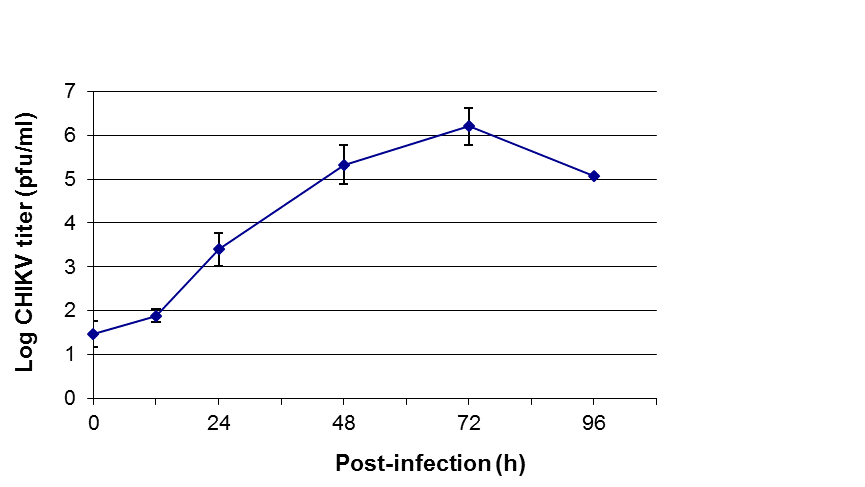


**Figure S2. Growth kinetic of CHIKV in HeLa CCL2 cells.** HeLa cells were seeded at 100% confluency and infected with CHIKV (GenBank; FJ445502) at M.O.I. 0.1. Viral supernatant was harvested at the indicated timepoint of post-infection and subjected to standard viral plaque assays. Error bars are indicative of mean ± s.e.m. expressed from three independent set of experiments.
